# HDAC Inhibitor Sodium Butyrate Attenuates the DNA Repair in Transformed but Not in Normal Fibroblasts

**DOI:** 10.3390/ijms23073517

**Published:** 2022-03-23

**Authors:** Olga O. Gnedina, Alisa V. Morshneva, Elena V. Skvortsova, Maria V. Igotti

**Affiliations:** Institute of Cytology, Russian Academy of Sciences, 194064 St. Petersburg, Russia; 1195alisa@gmail.com (A.V.M.); evavdeeva@mail.ru (E.V.S.); marie.igotti@gmail.com (M.V.I.)

**Keywords:** DNA damage response, DSB repair, histone deacetylase inhibitor (HDACi), cancer, MRN complex

## Abstract

Many cancer therapy strategies cause DNA damage leading to the death of tumor cells. The DNA damage response (DDR) modulators are considered as promising candidates for use in combination therapy to enhance the efficacy of DNA-damage-mediated cancer treatment. The inhibitors of histone deacetylases (HDACis) exhibit selective antiproliferative effects against transformed and tumor cells and could enhance tumor cell sensitivity to genotoxic agents, which is partly attributed to their ability to interfere with DDR. Using the comet assay and host-cell reactivation of transcription, as well as γH2AX staining, we have shown that sodium butyrate inhibited DNA double-strand break (DSB) repair of both endo- and exogenous DNA in transformed but not in normal cells. According to our data, the dysregulation of the key repair proteins, especially the phosphorylated Mre11 pool decrease, is the cause of DNA repair impairment in transformed cells. The inability of HDACis to obstruct DSB repair in normal cells shown in this work demonstrates the advantages of HDACis in combination therapy with genotoxic agents to selectively enhance their cytotoxic activity in cancer cells.

## 1. Introduction

Chemical and radiation therapies remain the most frequently used anticancer strategies, aiming at causing extensive DNA damage. This strategy relies on the intensive proliferation of cancer cells in comparison to the slow division of healthy cells [1]. The DNA damage leads to various genomic rearrangements, including deletions, amplifications, insertions, inversions, and translocations, which are believed to be among the drivers of carcinogenesis [2]. DNA damage activates the processes that are necessary to maintain genome integrity and cell survival, including cell cycle arrest and DNA repair. Cancer cells modify the DNA damage response system that normally protects cells from genomic instability [3]; thereby, the tumor becomes more sensitive to the genotoxic agents. Therefore, many chemotherapeutic agents directly or indirectly target the regulation of DNA integrity and lead to DNA damage and cell apoptosis. Despite the widespread use of genotoxic agents in cancer treatment, the development of drug resistance and high toxicity is common [4,5]. Accordingly, it is necessary to increase the effectiveness of genotoxic drugs and reduce the dosage. The combined use of DNA damaging agents with a substance that enhances the cytotoxic effect selectively in tumor cells is a promising approach.

Various DNA lesions occur in cells, and double-strand breaks (DSBs) are the most severe since they can result in the loss of genetic material and cell death. The cell’s reaction to the DNA damage, named DNA damage response (DDR), includes damage recognition, cell cycle checkpoint activation, DNA damage repair, and induction of cellular senescence or apoptosis in case of irreparable damage. DSBs are detected by the DNA damage signaling machinery, and the majority are repaired by either homologous recombination (HR) or non-homologous end-joining (NHEJ). The DDR is activated by the binding of the MRN complex consisting of Mre11/Rad50/Nbs1 proteins at the DNA damage site [6]. MRN tethers the broken DNA ends together [7] and recruits ATM kinase to damaged sites [8], which allows further DDR signaling. The MRN complex is involved in many stages of the DDR. Thus, besides the recognition of the DSB and the binding of broken DNA ends, the nuclease activity of the Mre11 protein within the MRN complex is required for both major DSB repair mechanisms, i.e., HR and NHEJ [9,10,11,12,13].

NHEJ is an error-prone DNA repair mechanism, which is active throughout the cell cycle but is strictly preferred in the G1/early S-phase [14,15]. Canonical NHEJ (c-NHEJ) starts with detection of the DSB by the Ku70/80 heterodimer and followed by the binding of the DNA ends using the Ku/DNA-PKcs complex [16,17]. The Ku70/Ku80 heterodimer interacts with most NHEJ repair factors and recruits them to the DSB site [18], determining the choice of the DNA repair pathway. Resection-dependent NHEJ (rd-NHEJ) and microhomology-mediated end joining (MMEJ) require the nuclease activity of Mre11 protein [12,13].

Mre11 nuclease activity plays a central role in DSB repair pathway choice. Mre11 nuclease activity promotes DSB end resection, committing to HR [19], and counteracts Ku binding, preventing c-NHEJ [20].

Homologous recombination is a high-fidelity DNA repair pathway that is restricted to the S and G2 phases of the cell cycle due to the cell-cycle-dependent availability of sister chromatids. The central factor of HR, Rad51 recombinase, forms nucleoprotein filaments at the ends of single-stranded DNA to establish correspondence and exchange between damaged and intact homologous DNA molecules [21].

Despite the increased expression of several DNA repair genes in tumors, proteins that are engaged in DDR [22] and DNA repair pathways [23] are often mutated in malignant cells, leading to aberrant DNA repair. In some cases, tumor cells overcome numerous DNA damages, decreasing the efficiency of high-fidelity repair pathways and exploiting more error-prone mechanisms for compensation, which increases genome instability and tumor progression [24]. On the one hand, DDR breakdown leads to damage accumulation and genome instability, and on the other hand, tumor cells become more dependent on the remaining signal pathways and more sensitive to chemotherapy. Therefore, the efficiency of genotoxic therapy may be increased through deregulation of DNA damage response and repair mechanisms. DDR modulation is seen as a promising strategy for anticancer therapy adaptation.

Histone deacetylase inhibitors (HDACis) can be considered as promising candidates for combined anticancer therapy [25]. HDACis exhibit an antiproliferative effect in a wide range of oncogene-transformed and cancer cells, causing cell cycle arrest, premature cell senescence, and programmed cell death [26]. HDACis have low toxicity in normal, noncancerous cells [27,28,29,30]. The use of HDACis as monotherapy has been effective in preclinical studies, but clinical trials have had limited success. However, combination therapy with other anticancer drugs has shown synergistic effects in both preclinical and clinical studies, demonstrating an increased susceptibility of tumor cells to the used drugs, i.e., sensitization of tumor cells [25]. Except for their antiproliferative effect, HDACis can sensitize cancer cells to genotoxic therapy and cause persistence of γH2AX foci [28,31,32] due to DNA damage response modulation. At the same time, HDACis are unable to radiosensitize normal human lung fibroblasts [30,33], suggesting their selectivity toward cancer cells. HDACis are reported to regulate the DNA damage response through a weakening of stress-induced expression of DDR and DNA repair proteins, such as ATM, BRCA1/2 [28,31], Ku70/Ku80, Rad51, and Mre11 [27,29,30,34,35], in cancer but not in normal cells [29,30]. However, further research is needed to determine the molecular basis of this phenomenon and the targets involved in HDACi-induced sensitization of cancer cells, as well as their potential protective effect on non-cancerous cells.

In order to identify the molecular mechanisms of selective sensitization of transformed cells, but not of normal cells, under the combined treatment with genotoxic agents and HDAC inhibitors, we compared the efficiency of DNA DSB repair in normal (MEF, mouse embryo fibroblasts) and oncogene-transformed cells (E1A+Ras-transformed MEF), treated with the HDACi sodium butyrate (NaBut), as well as the DNA repair system state. Up to 30% of all human tumors screened are found to carry mutations in any of the *ras* genes [4]. Therefore, studies aimed at increasing the efficiency of genotoxic anticancer therapy in cells with activated Ras seem to be especially relevant at present. Our results show that the HDACi sodium butyrate efficiently attenuates the DNA repair in transformed but not in normal cells due to selective dysregulation of the key repair proteins and destruction of the MRN (Mre11/Rad50/Nbs1) complex. These results indicate that the combination therapy of genotoxic agents and HDACis would be an attractive therapeutic strategy for selective enhancement of cytotoxic action in cancer cells.

## 2. Results

### 2.1. Histone Deacetylase Inhibitor Sodium Butyrate Prolongs Genotoxic-Induced DNA Double-Strand Breaks in E1A+Ras-Transformed Cells but Not in Normal Cells

**Figure 1:**
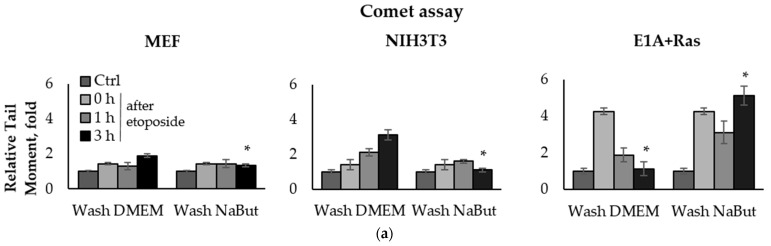

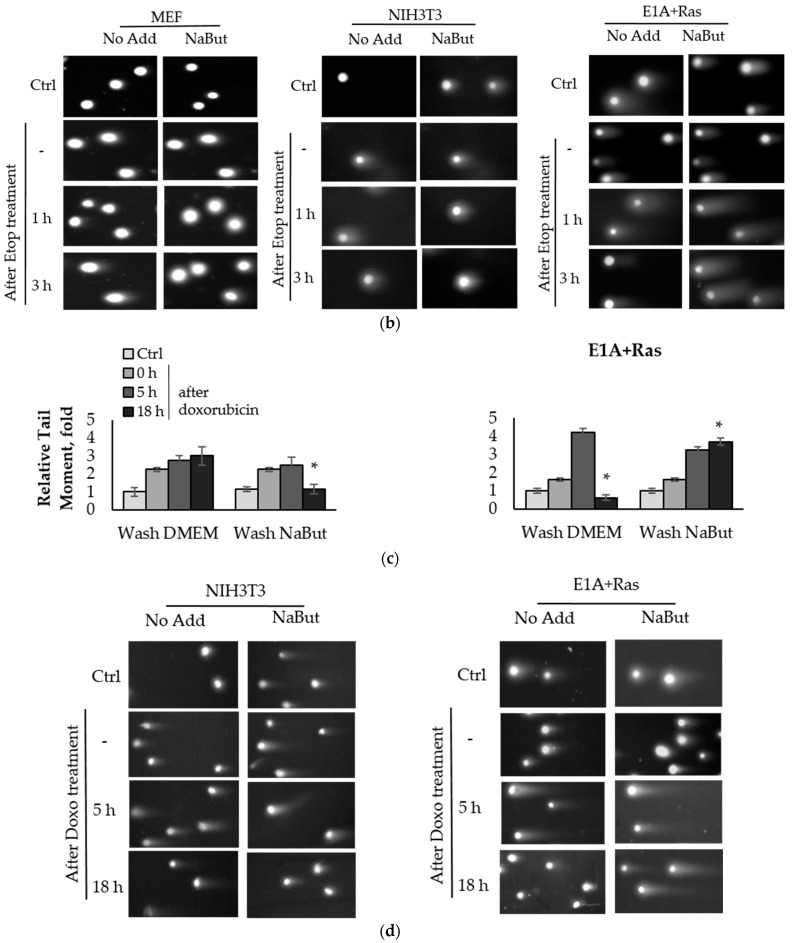
Sodium butyrate prolongs genotoxin-induced DNA double-strand breaks (DSBs) in oncogene-transformed cells but not in normal ones. Neutral comet assay data of non-transformed and transformed cells. Cells were untreated (Ctrl) or treated with 2.5 µM etoposide for 1 h (**a**) or 0.2 µg/mL doxorubicin for 40 min (**c**), then washed with or without sodium butyrate (NaBut) for 1–18 h. Comet assay data correspond to the relative number of DSBs and are represented as a mean of Olive tail moments, scaled to the untreated control (folds). Error bars indicate ± standard error of the mean. The statistical significance was determined using a Student’s *t* test (* = *p* < 0.05) between the Relative tail moment of the transformed vs. non-transformed doxorubicin/etoposide-treated cells washed with or without NaBut. (**b**,**d**) Representative images of experiments with etoposide (**b**) and doxorubicin (**d**). The images were obtained using an Axioskop, 10× lens.

To compare the effect of the HDACi sodium butyrate (NaBut) on the repair of damaged DNA in normal and oncogene-transformed cells, we used the comet assay method. E1A+Ras-transformed and normal mouse embryonic fibroblasts (MEF and NIH3T3) were treated with topoisomerase II inhibitors (etoposide, doxorubicin) for 1 h and then washed with DMEM in the absence or presence of NaBut. The damage state of endogenous DNA was assessed 1–18 h after the genotoxic treatment using the neutral comet assay, which allows the detection of DSBs by subjecting lysed cell nuclei to an electrophoretic field at neutral pH. The bar plots in Figure 1 represent the relative Olive tail moment parameter corresponding to the relative number of DSBs in a single cell. The comet assay data demonstrate a significant difference (*p*-value < 0.05) in the dynamics of DSB repair in transformed and normal cells (Figure 1).

The topoisomerase II inhibitors, etoposide and doxorubicin, induce DSBs through the cleavage complex stabilization during DNA replication [36], implying that the agents are effective only in actively proliferating cells. As expected, one-hour treatment with etoposide or doxorubicin does not cause a significant accumulation of the DNA breaks in slowly dividing MEF and NIH3T3 cells (Figure 1a,c). Etoposide and doxorubicin are chromatin intercalators [37,38], so they are able to inhibit topoisomerase II even after drug removal. Therefore, the number of DNA breaks increases after etoposide/doxorubicin removal in the absence of HDACis. In rapidly dividing E1A+Ras-transformed cells, genotoxic drugs cause a fourfold accumulation of DSBs, which are repaired with high efficiency (Figure 1a,c), followed by a decrease in the DSB number to the initial level after 3–18 h (Figure 1a,c). However, in the presence of NaBut, induced DSBs were unrepaired but persisted at a high level throughout the observation period (up to 18 h). Thus, our results demonstrate that the HDACi sodium butyrate strongly suppresses the DNA break repair in oncogene-transformed cells.

### 2.2. Sodium Butyrate Prolongs a Doxorubicin-Induced Persistence of the Phosphorylated Histone H2AX in Transformed Cells

Phosphorylation of histone H2AX is the first DNA damage marker, triggering the recognition and repair of DNA damages. The accumulation of phosphorylated H2AX foci (γH2AX) indicates the presence of numerous unrepaired DNA breaks.

**Figure 2:**
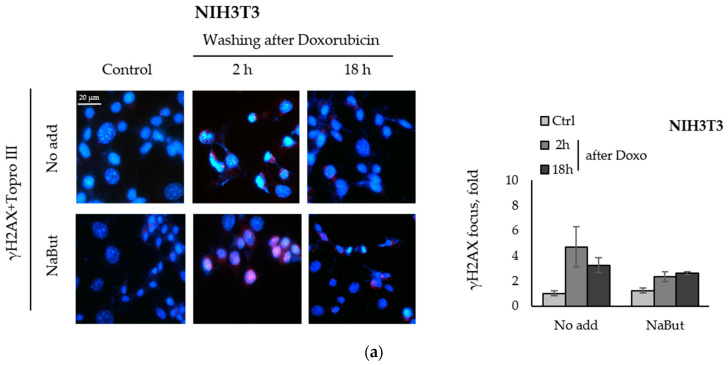

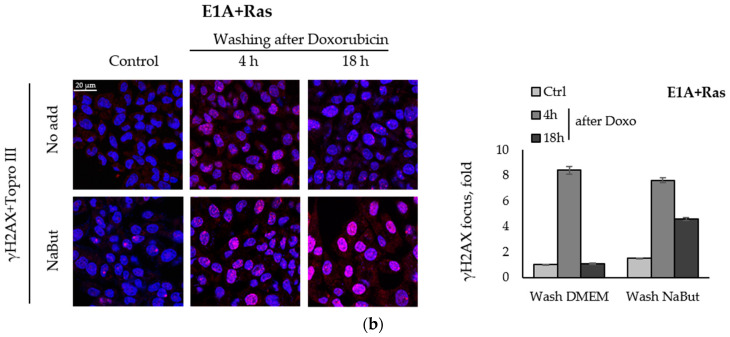
NaBut prolongs a doxorubicin-induced γH2AX foci persistence in transformed cells. Immunofluorescence analysis of γH2AX foci presence in non-transformed NIH3T3 cells (**a**) and in oncogene-transformed E1A+Ras cells (**b**). Cells were left untreated (Control, Ctrl) or treated with 0.2 µg/mL doxorubicin for 40 min and washed with DMEM with or without NaBut (NaBut/Ctrl) for 2–18 h. Cells were stained with rabbit anti-γH2AX antibodies, followed by Alexa-fluor 563 anti-rabbit (red) and Topro III (blue) staining. The images were obtained using an Olympus FV3000 microscope (40× lens). The integrated density of immunofluorescence data was evaluated using Image J © soft, scaled to the untreated control, and then plotted. Error bars indicate ± standard error of the mean.

We compared the effect of the HDACi sodium butyrate on the dynamics of γH2AX foci accumulation and disappearance in transformed and non-transformed cells treated with a genotoxic agent. E1A+Ras-transformed and non-transformed NIH3T3 cells were treated with topoisomerase II inhibitor doxorubicin for 40 min and then incubated with or without NaBut for 2–18 h. Immunofluorescence data show that treatment with sodium butyrate alone for 18 h did not cause the appearance of γH2AX nuclear staining in non-transformed NIH3T3 cells (Figure 2a). At the same time, γH2AX foci appeared in E1A+Ras-transformed cells treated with NaBut (Figure 2b). Doxorubicin-induced γH2AX foci disappeared within 18 h in both cell lines incubated in a NaBut-free medium. However, further treatment with NaBut led to the persistence of foci for up to 18 h only in transformed cells, but not in non-transformed NIH3T3 cells. We have previously shown that the HDACi sodium butyrate also enhances the accumulation of irradiation-induced γH2AX foci as well as their prolonged persistence [39].

Thus, our results indicate that NaBut prolongs the persistence of γH2AX foci induced by doxorubicin in transformed cells only.

### 2.3. The Histone Deacetylase Inhibitor Sodium Butyrate Inhibits the Repair of Exogenous DNA in Transformed Cells but Not in Normal Cells

To assess the influence of HDACis on the DNA repair process, we created a DNA repair model. To this end, a double-strand break was introduced into a luciferase gene of the pGL3-Luc reporter vector by the endonuclease. E1A+Ras, NIH3T3, and MEF cells were transfected by a linearized pGL3-Luc vector and then incubated with or without NaBut for 24–48 h. The DNA repair efficacy was estimated through the efficiency of the luciferase gene repair, performed by the DNA repair system of host cells (host cell reactivation of transcription assay, HCR assay).

**Figure 3:**
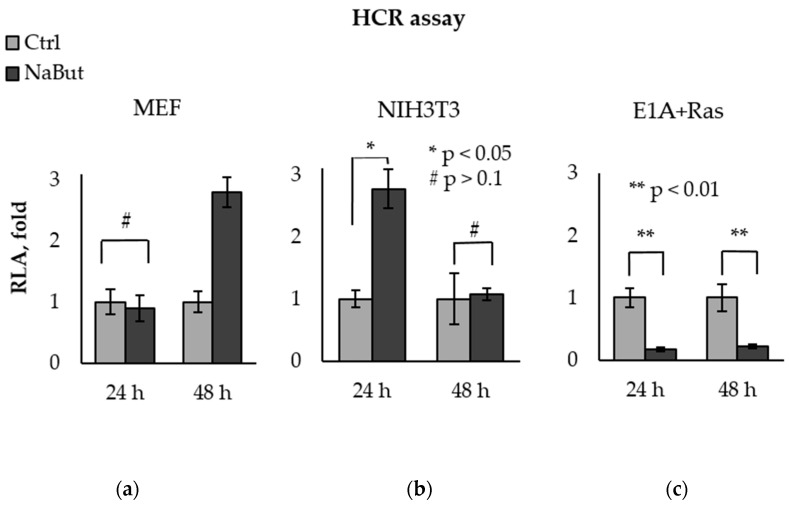
NaBut inhibits the repair of exogenous DNA in transformed cells. Host cell reactivation of transcription of the endonuclease-damaged pGL3-Luc in MEF (**a**), NIH3T3 (**b**), and E1A+Ras cells (**c**). Cells were transfected with intact or damaged pGL3-Luc vector and incubated without NaBut (Ctrl, light gray) or with (NaBut, dark gray) for 24–48 h. The relative luciferase activity (RLA) corresponding to the DNA repair efficiency was calculated as the luciferase activity in cells transfected with damaged plasmid. RLA values were scaled to the RLA of untreated cells (folds). The bar plots show the average value of the RLA ± SEM from 3 or 4 independent experiments. Statistical significance was determined using a Mann–Whitney U test (* = *p* < 0.05; ** = *p* < 0.01 # = *p* > 0.1), the comparison was performed between NaBut-treated and untreated cells.

The bar plots in Figure 3 present the relative luciferase activity (RLA), calculated as the activity of luciferase in cells with damaged pGL3-Luc. The luciferase activity in untreated cells was taken as a unit. Luciferase assay data reveal that NaBut was unable to decrease the recovery of the luciferase reporter in normal MEF cells, as well as in NIH3T3 cells, but significantly inhibited exogenous DNA repair in transformed E1A+Ras cells (Figure 3).

In order to exclude the specificity of the effect exclusively for NaBut, we investigated the effect of other HDAC inhibitors (SAHA and trichostatin A) on the efficiency of DNA repair in normal and oncogene-transformed cells. The HCR data show that, similar to sodium butyrate, SAHA and TSA inhibited the efficiency of DNA repair only in transformed E1A+Ras cells, but not in normal cells (Supplementary Figure S1).

Thus, we have shown that NaBut inhibits the repair of endogenous DNA damaged by etoposide/doxorubicin and also reduces the efficiency of exogenous DNA repair in transformed, but not in normal, cells.

### 2.4. NaBut Interferes with DNA Repair Machinery in Transformed Cells

To elucidate the molecular basis of NaBut-mediated modulation of the DSB repair system specifically in transformed cells, we compared the effect of NaBut on the repair protein expression in E1A+Ras-transformed and non-transformed MEF and NIH3T3 cells treated with NaBut for 48 h.

**Figure 4:**
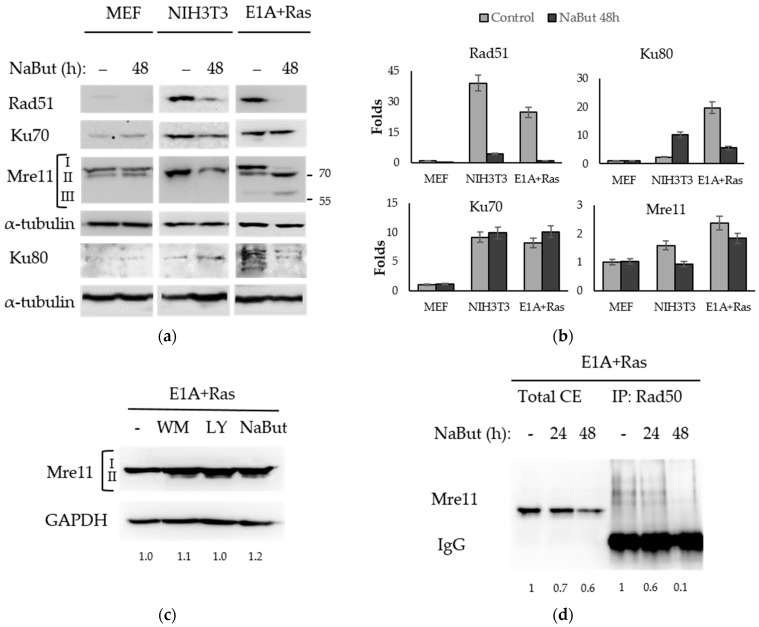
NaBut modulates the repair proteins in transformed cells. (**a**) Immunoblotting with antibodies to repair proteins using normal (MEF, NIH3T3) and transformed (E1A+Ras) cells untreated (−) or treated with 4 mM NaBut for 48 h. Immunoblotting with α-tubulin antibodies were used as a loading control. I—hyperphosphorylated Mre11, II—hypophosphorylated Mre11, III—truncated Mre11. (**b**) The bar plots represent band densities, evaluated using Image J © soft, scaled to the loading control, and normalized to the band density in untreated MEF cells (folds). For Ku80 and Mre11 proteins, the relevant plots display the aggregated intensity of all detected bands at each specific point, both for untreated and NaBut-treated cells. (**c**) Immunoblotting with antibodies to Mre11 using E1A+Ras cells untreated (−) or treated with 2.5 µM wortmannin (WM), 20 µM LY294002 (LY), or 10 mM NaBut for 8 h. GAPDH expression was used as a loading control. (**d**) Immunoblotting with antibodies to Mre11 using E1A+Ras cells untreated (−) or treated with NaBut for 24–48 h. Lanes 1–3 represent total cell extracts (total CE), and lanes 4–6 represent extracts co-precipitated with Rad50 antibodies.

Rad51 recombinase is the central protein of the HR pathway of DNA DSB repair. The immunoblotting data in Figure 4a reveal that NaBut decreased the amount of Rad51 protein both in transformed and non-transformed cells. However, the initial amount of Rad51 protein in transformed cells was significantly higher than that in normal MEF cells.

The Ku heterodimer (Ku70/Ku80) is a main component of the NHEJ repair pathway. NaBut does not affect the expression of Ku70 in all studied cell lines, but it causes a fivefold decrease in the Ku80 expression in transformed cells but not in normal MEF (Figure 4a,b). The different effect of NaBut on the Ku80 protein stability can be due to some differences between primary MEF cells (mouse embryonic fibroblasts) and spontaneously immortalized mouse fibroblasts NIH3T3. This phenomenon may be considered as a scope for further research.

Mre11 expression is slightly dependent on sodium butyrate (Figure 4a,b) in both normal and transformed cells. According to our RT-PCR data (see Supplementary Figure S2a), NaBut treatment does not decrease *mre11* gene expression in transformed cells. We have shown that HDACis control the Mre11 level, through the modulation of the Mre11 protein’s stability. In this way, in E1A+Ras-transformed cells, NaBut induced a redistribution of the modified forms of the Mre11 protein causing the disappearance of the upper fraction of Mre11 protein with an approximate mass of 80–90 kDa and the accumulation of the rapidly migrating Mre11 (Figure 4a), described in the literature as a hypophosphorylated form of Mre11 [40]. Moreover, NaBut induced the accumulation of another fast-moving Mre11 fraction with an approximate weight of 60 kDa (Figure 4a), described as a truncated Mre11 form, lacking nuclease activity and DNA-binding ability [41]. In order to find out whether the detected two forms of Mre11 protein differed in the Mre11 phosphorylation state, inhibitors of PI3-like kinases (wortmannin, LY294002) were used. Our data show that inhibition of PI3K led to the appearance of a rapidly migrating hypophosphorylated form of Mre11, which was also present under the NaBut treatment (Figure 4c).

Next, we investigated whether NaBut-induced Mre11 form redistribution led to the modulation of the MRN complex’s stability in E1A+Ras cells. The co-immunoprecipitation assay followed by immunoblotting revealed that prolonged NaBut treatment led to a weaking of the Mre11/Rad50 protein interaction in E1A+Ras transformants (Figure 4d).

Thus, despite the unchanging total amount of the Mre11 protein, its interaction with the MRN complex protein Rad50 weakened, suggesting the destruction of the MRN complex in transformed cells under the NaBut treatment.

## 3. Discussion

One of the important problems of anticancer treatment is the high toxicity of the used drugs toward normal cells. Combination therapy with agents that enhance the effect of cytostatics in transformed cells but have low toxicity toward normal cells is a promising strategy for tumor treatment. In this work, we studied the molecular mechanisms of preferential sensitization of transformed cells compared to normal cells under combined exposure to genotoxic agents and HDAC inhibitors.

We have shown that the HDACi sodium butyrate selectively attenuates the DSB repair in transformed cells and does not hamper the DNA repair ability in normal cells. Neutral comet assay revealed the successful DNA repair in E1A+Ras-transformed cells, which was abolished under NaBut treatment (Figure 1). This is in line with immunofluorescence data, demonstrating the disappearance of phospho-H2AX foci 18 h after genotoxic treatment in E1A+Ras-transformed cells. However, NaBut prolonged the persistence of phospho-H2AX foci up to 18 h (Figure 2). In this respect, we hypothesize that the HDAC inhibitor NaBut hinders the repair of genotoxic-induced DNA DSBs in oncogene-transformed cells.

To confirm the interference of NaBut in DSB repair, we performed a reactivation of damaged DNA transcription assay (HCR). The HCR assay revealed that NaBut suppressed the efficiency of DNA double-strand break repair in transformed E1A+Ras cells, whereas in normal cells (MEF, NIH3T3), NaBut did not reduce DNA repair.

Our data were reproduced in tumor and non-tumor human cells (data not shown). HCR analysis showed that NaBut reduced the repair efficiency of damaged endogenous DNA in HCT116 colon cancer cells but did not affect repair in non-cancerous HEK293 cells. This observation is in line with an earlier study [42], demonstrating that the HDACi sodium butyrate sensitized breast cancer cells MCF-7 to etoposide more effectively than non-cancerous HEK293 cells, provoking the accumulation of γH2AX foci and a decrease in cancer cell viability. However, in our study, we did not simply track the long-term effects of unrepaired breaks but rather used the direct DNA repair modeling through the host cell transcriptional reactivation assay to demonstrate HDACi-induced downregulation of DNA repair in transformed and cancer cells.

Looking for the mechanisms underlying the HDACi’s influence on DNA repair, we analyzed the effect of NaBut on the expression of repair proteins. Rad51 is a key protein of HR, taking place in the S-phase of the cell cycle. The observed HDACi-induced decrease in Rad51 is consistent with the findings of other authors [27,34], and it is likely due to the HDACi-induced cell cycle arrest. Thus, the *rad51* gene is the target of the E2F transcription factor [43], the activity of which decreases at HDACi-induced G1/S cell cycle arrest [44,45].

Increased basal Rad51 levels in transformed cells compared to those in normal MEF cells, shown in this study, are consistent with the current notion that Rad51 is often overexpressed in tumor cells, resulting in increased resistance to genotoxic therapy [46,47]. The high levels of Rad51 may also be associated with genome instability [48], suggesting that the NaBut-mediated decrease in Rad51 in transformed cells may lead to overcoming the tumor’s chemoresistance.

Next, we showed that NaBut differentially affected the expression of the critical NHEJ repair proteins Ku70 and Ku80 in the transformed cells. Thus, the expression of Ku70 did not depend on NaBut treatment, whereas the level of the Ku80 protein decreases by about fivefold under NaBut treatment of transformed cells. The expression of two Ku subunits estimated in several types of clinical samples was always in the ratio 1:2 (Ku80:Ku70) both in normal and tumor tissues [49], suggesting that Ku80 is a limiting factor for heterodimer formation. At the same time, in normal cells, the expression of Ku70 and Ku80 turned out to be independent of HDACis. Therefore, we assume that a Ku80 level decrease leads to a diminution of the Ku70/Ku80 efficiency and inhibition of NHEJ in transformed cells. The observed effect of HDACi on the key NHEJ repair proteins in transformed cells is consistent with other studies [30,35,50,51].

Finally, the Mre11 protein, which is required for both DSB repair mechanisms, was the focus of our attention. We have shown that NaBut did not affect the Mre11 expression in both normal and transformed cells. However, a redistribution of Mre11 forms was found upon NaBut treatment exclusively in oncogene-transformed cells (Figure 4a). NaBut has been shown to induce the disappearance of the slowly migrating hyperphosphorylated fraction of Mre11 protein and the accumulation of the rapidly migrating hypophosphorylated form, suggesting a NaBut-induced decrease in Mre11 phosphorylation in transformed cells. Besides the accumulation of the hypophosphorylated Mre11, the appearance of the truncated Mre11 protein was demonstrated. To elucidate the proteasome degradation pathway’s role in the Mre11 truncation, a proteasome inhibitor, lactacystin (LC), was used. According to our data (Supplementary Figure S2b), both LC alone and in combination with NaBut resulted in the accumulation of TR-Mre11 in E1A+Ras cells, implying that truncation of full-length Mre11 to TR-Mre11 is independent of the proteasome pathway. Rather, the proteasome pathway is involved in further degradation of TR-Mre11, since TR-Mre11 was observed to be stabilized by LC. It was observed that the NaBut-induced redistribution of Mre11 protein forms was accompanied by a decrease in the Mre11 and Rad50 protein interaction in transformed E1A+Ras cells (Figure 4d).

The Mre11 protein activity can be regulated by various post-translational modifications, including phosphorylation, methylation, ubiquitination, and even protein truncation [52]. The hyperphosphorylation of Mre11 occurs upon DNA damage and precedes MRN complex assembly [53]. Therefore, Mre11 dephosphorylation may be a reason for the dissociation of the Mre11/Rad50/Nbs1 complex. HDACis induce the accumulation of hypophosphorylated Mre11 associated with unrepaired DNA breaks but impair the interaction of Mre11 with Rad50. The dissociation of Mre11/Rad50 could contribute to the sensitization of transformed cells to genotoxic stress, as an unfunctional MRN complex leads to the defective recognition and tethering of DNA double-strand breaks. This is in line with studies of the O’Malley Jr and Li group [54,55], in which MRN complex disruption was shown to mediate tumor sensitivity to cisplatin.

Different kinases are responsible for Mre11 phosphorylation [52]. In most cases, hyperphosphorylation of the Mre11 protein correlates with the release of the MRN complex from damaged DNA, irrespective of the exact kinase pathway. The phosphorylation of Mre11 protein (Ser676/Ser678) by PIKK family kinases, in particular, ATM kinase, leads to a weakening of the DNA-binding ability of Mre11 and serves as a feedback mechanism, preventing excessive nuclease activity of Mre11 [40]. The Rsk-dependent phosphorylation of Mre11 (Ser676) inhibits Mre11 binding to DSB [56] also. As a consequence, there is a high correlation between Raf/MAPK/Rsk pathway activation and tumor radioresistance [56]. Phosphorylation of Mre11 (Ser649) by Plk1 kinase during DDR is the priming for subsequent Mre11 phosphorylation by CK2 kinase (Ser688) [57]. CK2-mediated Mre11 phosphorylation, as well as ATM-dependent phosphorylation, inhibits the MRN complex loading into the DSB site, resulting in decreased DNA repair [57]. In view of the above, it can be assumed that HDACi-induced Mre11 dephosphorylation may promote the accumulation of the DNA-bound form of Mre11, which leads to DDR signaling disorder in transformed cells treated with HDACi. It appears that HDACi reduces Mre11 phosphorylation by inhibiting kinases rather than by activating phosphatases. HDACis are known to disrupt the interaction between HDAC and protein phosphatases resulting in attenuated dephosphorylation of target proteins [58,59]. Moreover, we have shown that the effect of NaBut is comparable to that caused by inhibitors of PI3-like kinases (Figure 4c).

The catalytic activity of CK2 is shown to increase as a result of tyrosine phosphorylation [60]. Our unpublished data reveal that NaBut reduces the Tyr phosphorylation of CK2 kinase in E1A+Ras-transformed cells. Accordingly, it can be assumed that NaBut decreases Mre11 phosphorylation in transformed cells through the suppression of CK2 activity caused by NaBut-dependent decline of CK2 Tyr-phosphorylation. As a result, hypophosphorylated Mre11 dissociates from the R2TP chaperone complex unit that leads to Mre11 protein destabilization [61]. Therefore, NaBut seems to be responsible for Mre11 protein truncation through the modulation of CK2 activity in transformed cells.

Accumulation of the truncated Mre11 form found in NaBut-treated transformed cells (Figure 4a) correlates with the results obtained in bladder cancer cells [41]. Nicholson et al. showed that the HDACi panobinostat caused the cleavages of the full-length Mre11 protein (80 kDa) and the accumulation of truncated Mre11 lacking the C-terminal region with a DNA-binding domain (approximate mass of 60 kDa). Lack of the Mre11 C-terminal decreased DNA repair efficiency, due to abolished recruitment of Mre11 to DSB, which consequently led to the radiosensitization of bladder cancer cells [41,62]. According to our unpublished data, NaBut is also able to increase the cytotoxic effect of DNA-damaging agents (etoposide, doxorubicin, irradiation) in cells transformed with oncogenes, which apparently is a consequence of the DNA repair efficiency inhibition. Therefore, the NaBut-induced suppression of the DNA repair in transformed cells is supposed to be partially mediated by an accumulation of truncated, aberrant Mre11 form that is unable to bind DNA and function as a nuclease.

In conclusion, our data suggest that the HDACi sodium butyrate selectively attenuates the DSB repair in transformed cells through the deregulation of the key repair proteins, mostly through Mre11 form redistribution in transformed cells. Our data suggest that NaBut inhibits Mre11 activity in at least two ways. First, NaBut-induced dephosphorylation of Mre11 could cause the Mre11/Rad50 interaction to decrease, followed by the dissociation of the MRN (Mre11/Rad50/Nbs1) complex. Second, cleavage of full-length Mre11 and accumulation of the truncated Mre11 form can lead to a weakening of the DNA-binding ability of Mre11 and the release of the MRN complex from DSB sites. NaBut is described to inhibit a wide range of histone deacetylases. However, the identification of the specific HDAC that is responsible for the sensitization of cancer cells could be an interesting topic for future research.

Our findings are important from the perspective of using HDACis in combined cancer therapy with genotoxic agents or irradiation. However, its clinical implication needs to be further tested since the role of the DNA repair system in cancer cell viability is ambiguous. It is widely discussed that activated DNA repair pathways allow cancer cells to recover from the DNA damage caused by anticancer drugs, which is one of the reasons for chemoresistance [63]. In this regard, anticancer therapy should include agents suppressing DNA repair to make cancer cells more sensitive to the DNA-damaging drugs. However, an important point of view has been accumulated, regarding drug-induced elimination of the majority of the tumor population, which is accompanied by the persistence of drug-tolerant dormant (quiescent) cancer cells [64], which are even more dangerous considering possible tumor relapse. In this context, unrepaired DSBs lead to the constitutive activation of ATM kinase, which in turn triggers the downstream NF-kB- and STAT3-mediated survival pathways, resulting in cancer stemness maintenance and post-therapy repopulation of the tumor [65]. This fact must be kept in mind in an attempt to restrain DNA repair and fuel DSB accumulation to increase the efficiency of anticancer therapy using genotoxic agents. Therefore, further studies need to be performed to determine how inhibition of DSB repair by HDACi treatment will affect cancer cells’ destiny in their native tumor microenvironment. However, the inhibition of DNA repair has proven to be highly effective in combined treatment according to numerous clinical trials [66,67], and the proposed combination with HDACi was aimed to decrease toxic side effects for cancer patients, increasing the effectiveness of genotoxic drugs and targeting only cancer cells.

## 4. Materials and Methods

### 4.1. Cell Lines

For this research, we used normal mouse embryonic fibroblasts (MEFs) at passages 2–5 and NIH3T3 cells obtained from the shared research facility “Vertebrate cell culture collection” supported by the Ministry of Science and Higher Education of the Russian Federation (Agreement no. 075-15-2021-683). E1A+Ras transformed cells were established from primary embryonic fibroblasts by Ca-phosphate transfection [68]. E1A+Ras and NIH3T3 cells were cultured in high-glucose Dulbecco’s modified Eagle’s medium (DMEM) with the addition of 10% fetal bovine serum (FBS) and antibiotics. For the primary cell line MEF, DMEM/F12 medium was used with the addition of 15% FBS and antibiotics. Cells were treated with HDAC inhibitor of class I and IIA sodium butyrate (NaBut, 4 mM) for 24–72 h (Merck, Darmstadt, Germany). The endogenous DNA was damaged by two topoisomerase II inhibitors (2.5 μM etoposide and 0.4 μg/mL doxorubicin—Merck, Darmstadt, Germany), whereas the exogenous plasmid DNA was damaged by the restriction endonuclease BglII (Thermo Fisher Scientific, Waltham, MA, USA).

### 4.2. Comet Assay

Cells were treated with the doxorubicin for 40 min at 0.4 µg/mL, and then washed with or without 4 mM NaBut for 5–18 h. Samples were collected at different time points and processed for neutral comet assay, evaluating DSB only [69]. For this, the pellet of cells (10^5^ cells) was resuspended by pipetting with 200 μL of 0.7% LMP agarose/PBS, at 37 °C. Then, 65 μL of agarose containing the cells was immediately transferred onto glass slides precoated with 1% agarose, covered with a 24 × 32 mm coverslip and placed on an ice-pack for solidification for 5–10 min. The coverslip was removed, and another 80 µL of LMP agarose was applied, covered with a 24 × 32 mm coverslip and placed at 4 °C for 10 min. After gelling, the coverslips were removed. Slides were submerged in lysis solution (2.5 M NaCl, 0.1 M EDTA, 10 mM Tris, pH 10, 1% N-laurylsarcosine, 0.5% Triton X-100) and incubated overnight at 4 °C in the dark. Slides were washed three times for 5 min with the electrophoresis buffer TBE (90 mM Tris base, 90 mM boric acid, 2 mM EDTA-Na2), slides were subjected to electrophoresis in TBE solution for 1 h at 1 V/cm at 4 °C. Cells were stained with propidium iodide (3 µg/mL) for 10 min and analyzed with a fluorescence microscope (Axioscope, Carl Zeiss, Oberkochen, Germany), 10× lens. At least 100 comet images per sample were analyzed. TriTek CometScore Software (v.2.0, Bioz, Los Altos, CA, USA) was used to evaluate Comet images to obtain the Olive tail moment parameter.

### 4.3. Host-Cell Reactivation Assay (HCR)

E1A+Ras-transformed fibroblasts and NIH3T3 cells were seeded in 96-well plates at a seeding density of 30 × 10^3^ cells per well in antibiotic-free DMEM with 10% FBS. A luciferase reporter vector pGL3-Luc was damaged with endonuclease and then introduced into cells using the Lipofectamine-2000 (Invitrogen, Waltham, MA, USA) transfection, as recommended by the manufacturer. After transfection, cells were incubated in the presence or absence of sodium butyrate (NaBut, 4 mM) for 24 or 48 h. Afterward, cells were lysed, and the DNA repair efficiency was evaluated by measurement of the luciferase activity, using the Luciferase assay system and Turner Designs luminometer (Promega, Madison, WI, USA). The relative luciferase activity (RLA) was calculated as a luciferase activity in cells with damaged plasmid, scaled to this one in untreated cells. Host-cell reactivation assay results are represented as a mean of relative luciferase activity (3–4 independent experiments), error bars indicate ±standard error of the mean.

### 4.4. Immunoblotting

Cells were lysed in PBS containing 1% NP-40, 0.5% sodium deoxycholate, 0.1% SDS, 20 mM glycerophosphate, 1 mM sodium orthovanadate, 5 mM EGTA, 10 mM sodium fluoride, 1 mM phenylmethylsulfonyl fluoride and protease inhibitor cocktail. The samples were equalized according to the amount of protein, which was measured spectrophotometrically at a wavelength of 595 nm according to the M. Bradford method [70]. Subsequently, 20–40 μg of total protein was used in different experiments. Samples were boiled in a loading buffer (60 mM Tris-Cl pH 6.8, 2% SDS, 10% glycerol, 5% β-mercaptoethanol, 0.01% bromophenol blue) at 100 °C for 5 min. Proteins were separated electrophoretically in 10–12.5% SDS-PAGE gel and transferred to a PVDF membrane. Membranes were blocked for 1.5 h at room temperature in blocking buffer (5% nonfat dry milk/PBST) and incubated with respective primary antibodies diluted in PBST for 1 h at room temperature or overnight at +4 °C. Then, membranes were incubated with HRP-conjugated secondary antibodies diluted in blocking buffer for 1 h at room temperature. As primary antibodies, we used antibodies to Mre11 #NB100-142 (1:5000), Ku80 #NB100-508 (1:500) (Novus Biologicals, Centennial, CO, USA), Ku70 #4588 (1:1000), Rad51 #8875 (1:1000) (Cell Signaling Technology, Danvers, MA, USA), GAPDH #2118 (1:1000) (Cell Signaling Technology, Danvers, MA, USA), and alpha-tubulin sc-32293 (1:10,000) (Santa Cruz Biotechnology, Dallas, TX, USA). The band density was evaluated using ImageJ soft (1.53e, NIH, Bethesda, MD, USA). Then, density values were scaled to loading control and were converted to relative units.

### 4.5. Immunoprecipitation

Cells were lysed in buffer, containing 10 mM Tris-HCl pH 7.4, 150 mM NaCl, 0.5% NP-40, 1% Triton X-100, 20 mM glycerophosphate, 1 mM sodium orthovanadate, 5 mM EGTA, 10 mM sodium fluoride, 1 mM phenylmethylsulfonyl fluoride and protease inhibitor cocktail. The samples were equalized according to the amount of protein, which was measured by the Bradford method, 500 µg of total protein was used. The lysates were incubated with Protein A agarose beads (1% bead slurry for one sample) at 4 °C for 1 h for the pre-clearing. The supernatant was transferred to the fresh tube and incubated with primary antibodies to Rad50 (1:300) (NB100-154, Novus Biologicals, Centennial, CO, USA) with gentle rocking at 4 °C overnight. The next day, Protein A agarose beads were added to the mixture (2% bead slurry for one sample) and incubated with gentle rocking at 4 °C for 1–3 h. After microcentrifuge for 5 min at 200× *g*, 4 °C, the pellet was washed three times with cell lysis buffer, resuspended with sample buffer for SDS-PAGE, heated to 95–100 °C for 5 min, and centrifuged for 1 min at 14,000× *g*. Then protein electrophoresis and immunoblotting were carried out, as described in the previous section. As primary antibodies, we used antibodies to Mre11 #NB 100-142 (Novus Biologicals, Centennial, CO, USA) (1:5000).

### 4.6. Immunofluorescence

The cells were seeded on cover glasses and then fixed at 3.7% PFA for 15 min at room temperature, washing with 0.15 M glycine solution for 15 min. After fixation, cells were permeabilized with 0.2% Triton X-100 and blocked using 3% BSA/PBS for 1.5 h. Cells were incubated with primary antibodies to phosphorylated H2AX (#2577, Cell Signaling Technology, Danvers, MA, USA), diluted 1:150 in the blocking buffer, at 4 °C overnight. Then cells were stained with Alexa Fluor 488–conjugated goat–anti rabbit secondary antibodies (1:500) for 1 h at room temperature in the dark. Simultaneously, nuclear staining with 1 mM TO-PRO^®^-3 was carried out. The images were obtained, using an Olympus FV3000 microscope (40× lens). The images with split channels are shown in the Supplementary Figure S3, as well as negative control. The immunofluorescence of about 200–250 cells was analyzed with ImageJ © soft according to the author’s custom protocol. Nuclear area was selected based on the blue channel signal (DAPI) to count cells, and the red channel signal (γH2AX) was measured selectively in the nuclear area to exclude the background signal. The integrated intensity of γH2AX was calculated as signal intensity per nucleus (the ratio of the total nuclear signal to the number of nuclei), scaled to the untreated control, and then plotted. Error bars indicate ±standard error of the mean.

## Supplementary Materials

The following supporting information can be downloaded at www.mdpi.com/article/10.3390/ijms23073517/s1.
