# Supplementary material for: HDAC Inhibitor Sodium Butyrate Attenuates the DNA Repair in Transformed but Not in Normal Fibroblasts
Source: Int J Mol Sci. 2022 Mar 23;23(7):3517. doi: 10.3390/ijms23073517 (PMC8998589; doi:10.3390/ijms23073517)
Supplement: Supplementary file 1 [file ijms-23-03517-s001.zip › ijms-1590176-supplementary.pptm]

## Slide 1
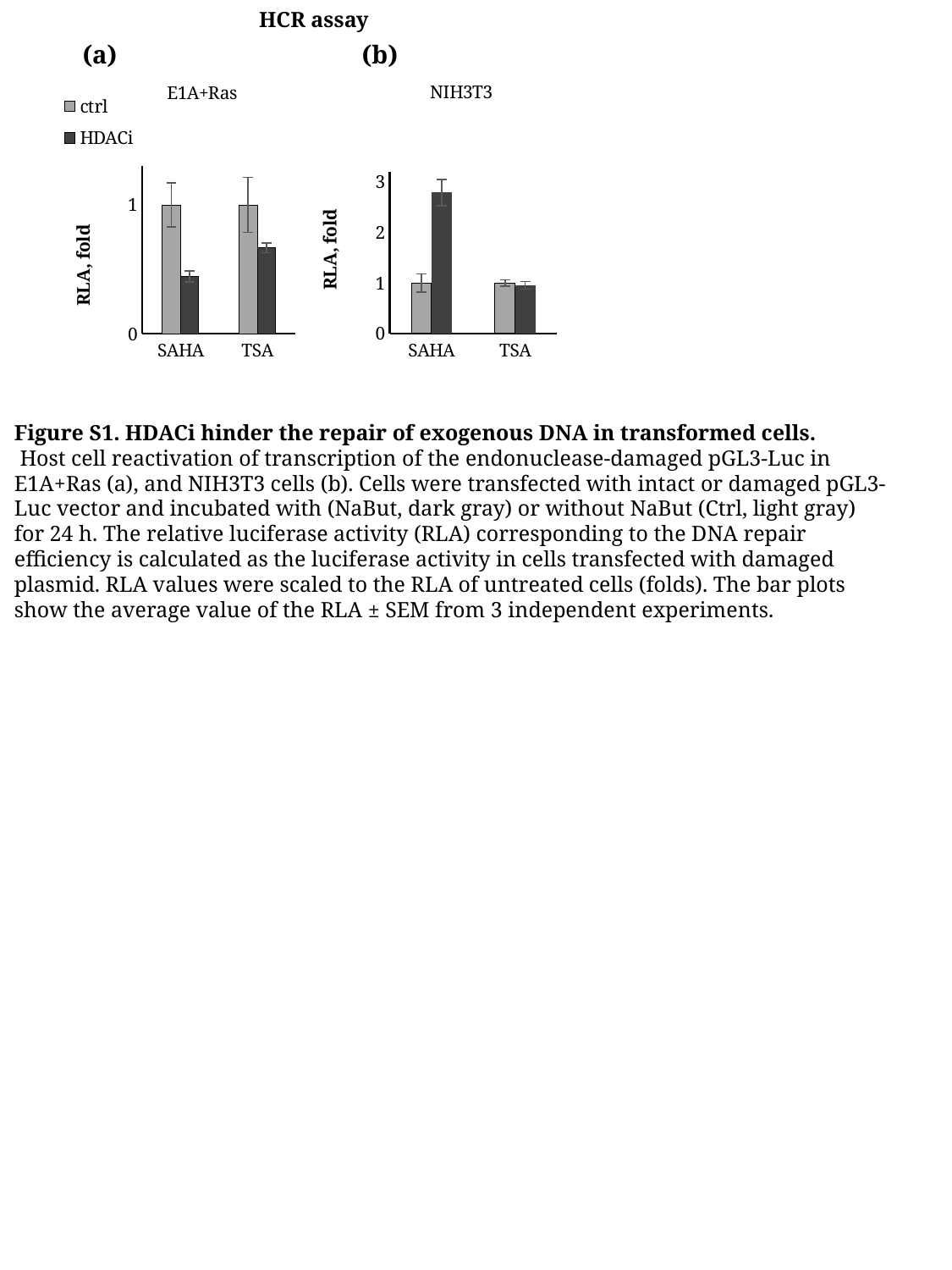

HCR assay
(a)
(b)
### Chart: E1A+Ras
| Category | ctrl | HDACi |
|---|---|---|
| SAHA | 1.0 | 0.4441940532081377 |
| TSA | 1.0 | 0.6674514816185125 |
### Chart: NIH3T3
| Category | ctrl | HDACi |
|---|---|---|
| SAHA | 1.0 | 2.789542700835584 |
| TSA | 1.0 | 0.9505878071266473 |Figure S1. HDACi hinder the repair of exogenous DNA in transformed cells.
 Host cell reactivation of transcription of the endonuclease-damaged pGL3-Luc in E1A+Ras (a), and NIH3T3 cells (b). Cells were transfected with intact or damaged pGL3-Luc vector and incubated with (NaBut, dark gray) or without NaBut (Ctrl, light gray) for 24 h. The relative luciferase activity (RLA) corresponding to the DNA repair efficiency is calculated as the luciferase activity in cells transfected with damaged plasmid. RLA values were scaled to the RLA of untreated cells (folds). The bar plots show the average value of the RLA ± SEM from 3 independent experiments.

## Slide 2
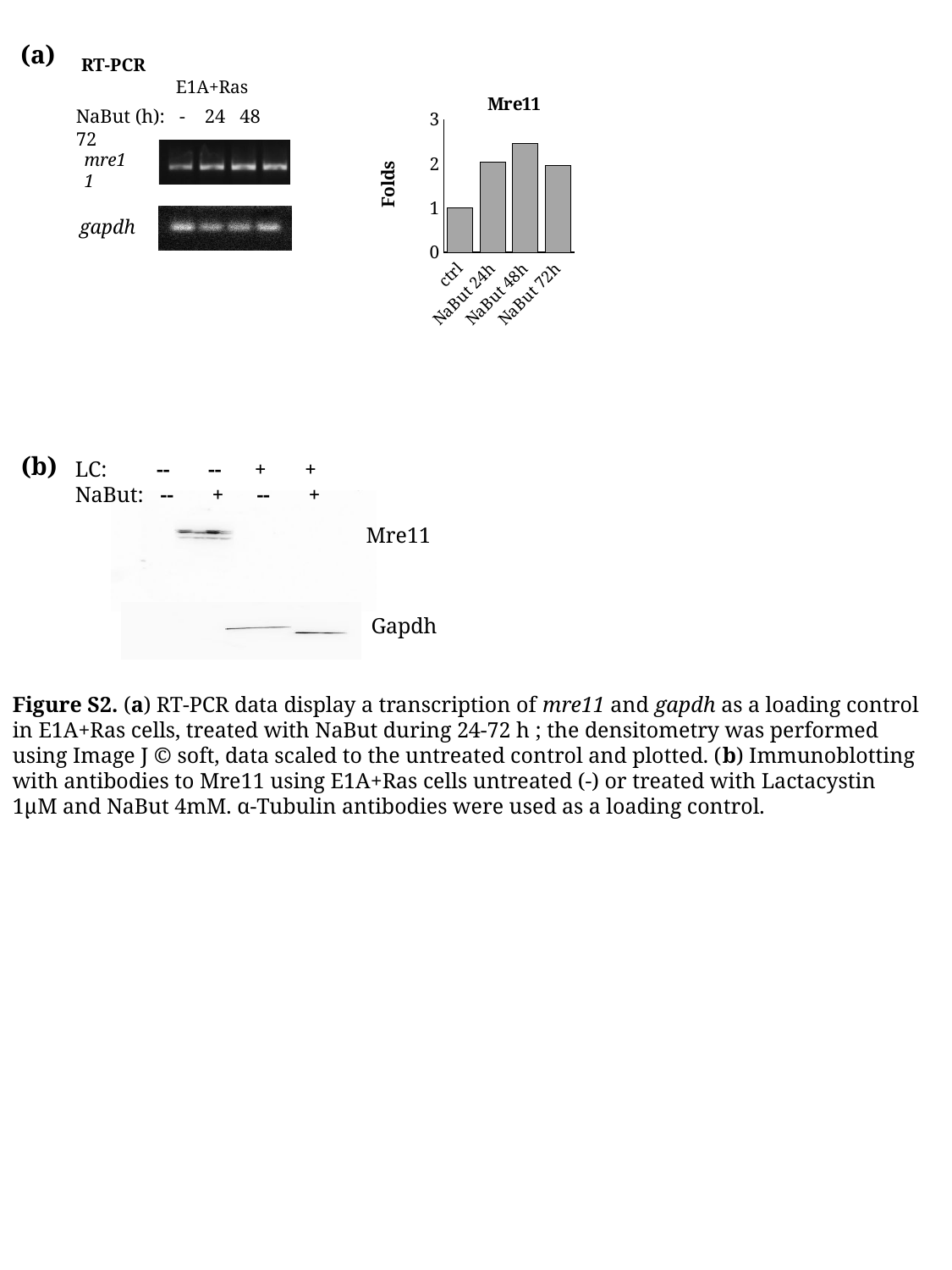

(a)
RT-PCR
### Chart:
| Category | Mre11 |
|---|---|
| ctrl | 1.0 |
| NaBut 24h | 2.034673411925124 |
| NaBut 48h | 2.4589868758819744 |
| NaBut 72h | 1.9692477352299305 |E1A+Ras
NaBut (h): - 24 48 72
mre11
gapdh
(b)
LC: -- -- + +
NaBut: -- + -- +
Mre11
Gapdh
Figure S2. (a) RT-PCR data display a transcription of mre11 and gapdh as a loading control in E1A+Ras cells, treated with NaBut during 24-72 h ; the densitometry was performed using Image J © soft, data scaled to the untreated control and plotted. (b) Immunoblotting with antibodies to Mre11 using E1A+Ras cells untreated (-) or treated with Lactacystin 1µM and NaBut 4mM. α-Tubulin antibodies were used as a loading control.

## Slide 3
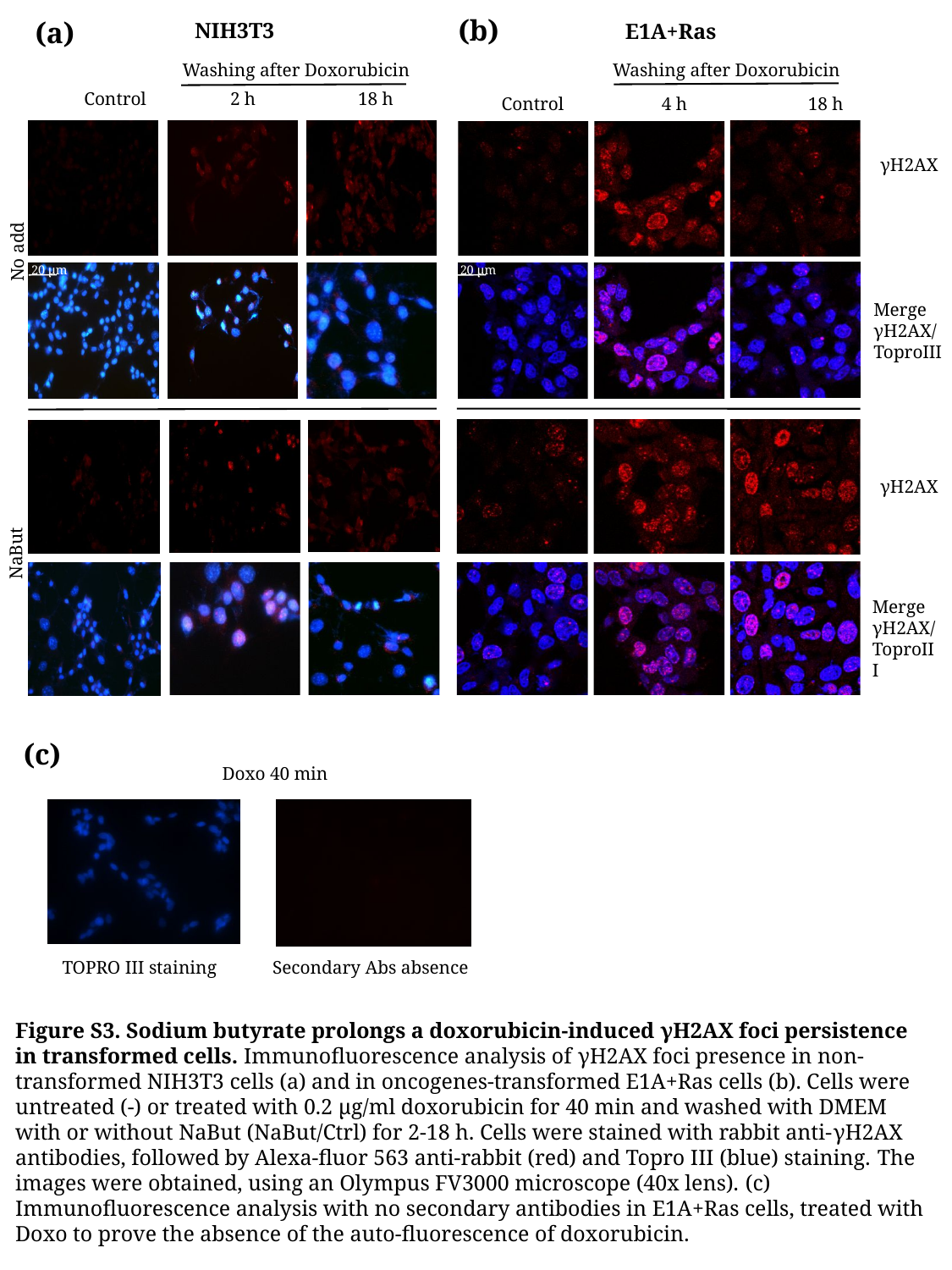

(b)
(a)
NIH3T3
E1A+Ras
Washing after Doxorubicin
Washing after Doxorubicin
 Control 2 h 18 h
 Control 4 h 18 h
γH2AX
20.00 μm
No add
20 µm
20 µm
Merge
γH2AX/
ToproIII
γH2AX
NaBut
Merge
γH2AX/
ToproIII
(c)
Doxo 40 min
TOPRO III staining Secondary Abs absence
Figure S3. Sodium butyrate prolongs a doxorubicin-induced γH2AX foci persistence in transformed cells. Immunofluorescence analysis of γH2AX foci presence in non-transformed NIH3T3 cells (a) and in oncogenes-transformed E1A+Ras cells (b). Cells were untreated (-) or treated with 0.2 µg/ml doxorubicin for 40 min and washed with DMEM with or without NaBut (NaBut/Ctrl) for 2-18 h. Cells were stained with rabbit anti-γH2AX antibodies, followed by Alexa-fluor 563 anti-rabbit (red) and Topro III (blue) staining. The images were obtained, using an Olympus FV3000 microscope (40x lens). (с) Immunofluorescence analysis with no secondary antibodies in E1A+Ras cells, treated with Doxo to prove the absence of the auto-fluorescence of doxorubicin.

## Slide 4
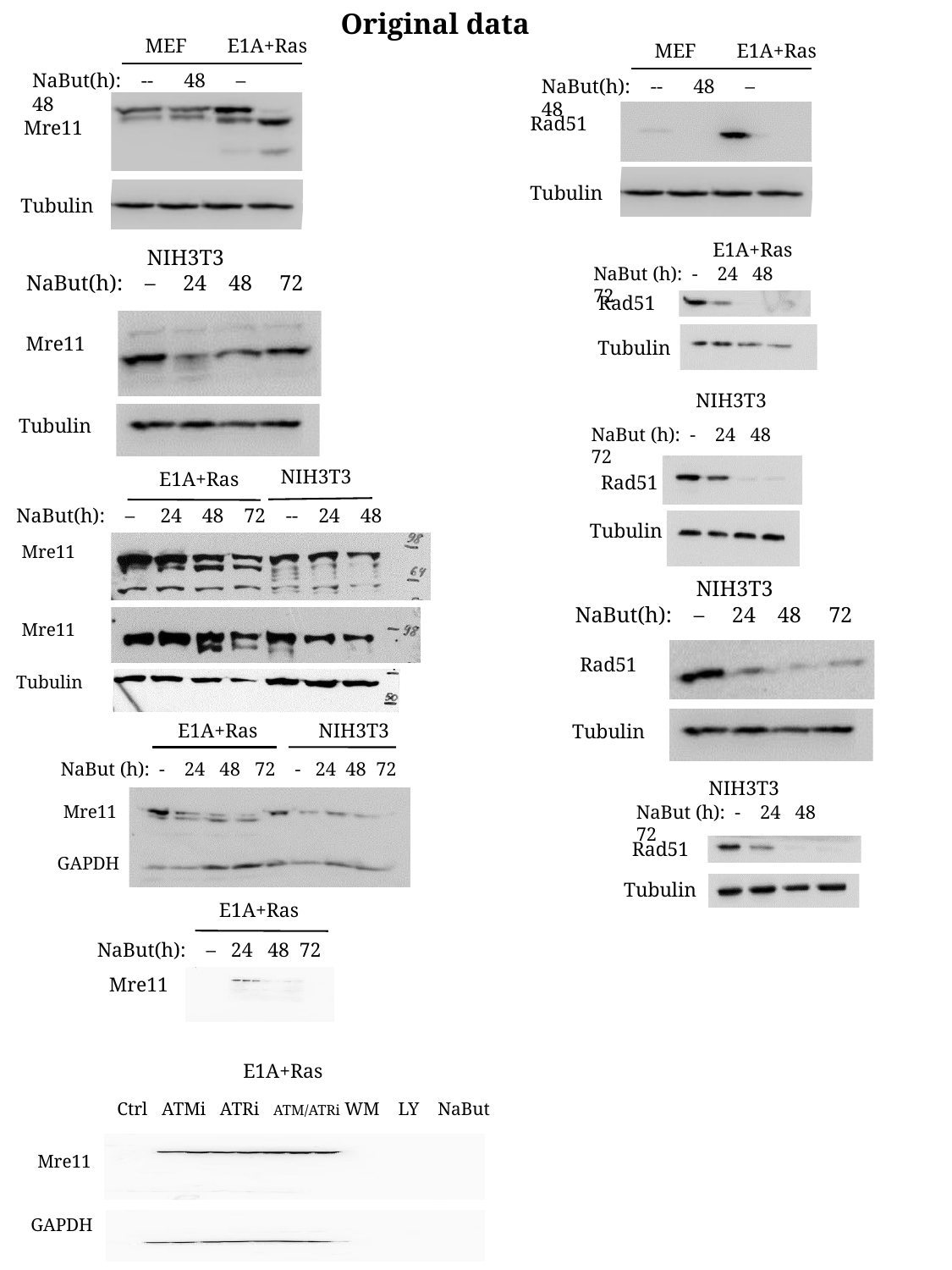

Original data
 MEF E1A+Ras
 MEF E1A+Ras
NaBut(h): -- 48 – 48
NaBut(h): -- 48 – 48
Rad51
Mre11
Tubulin
Tubulin
 E1A+Ras
 NIH3T3
NaBut(h): – 24 48 72
NaBut (h): - 24 48 72
Rad51
Mre11
Tubulin
NIH3T3
Tubulin
NaBut (h): - 24 48 72
NIH3T3
E1A+Ras
Rad51
NaBut(h): – 24 48 72 -- 24 48
Tubulin
Mre11
 NIH3T3
NaBut(h): – 24 48 72
Mre11
Rad51
Tubulin
 E1A+Ras NIH3T3
Tubulin
NaBut (h): - 24 48 72 - 24 48 72
NIH3T3
NaBut (h): - 24 48 72
Mre11
Rad51
GAPDH
Tubulin
E1A+Ras
 NaBut(h): – 24 48 72
Mre11
E1A+Ras
Ctrl ATMi ATRi ATM/ATRi WM LY NaBut
Mre11
GAPDH

## Slide 5
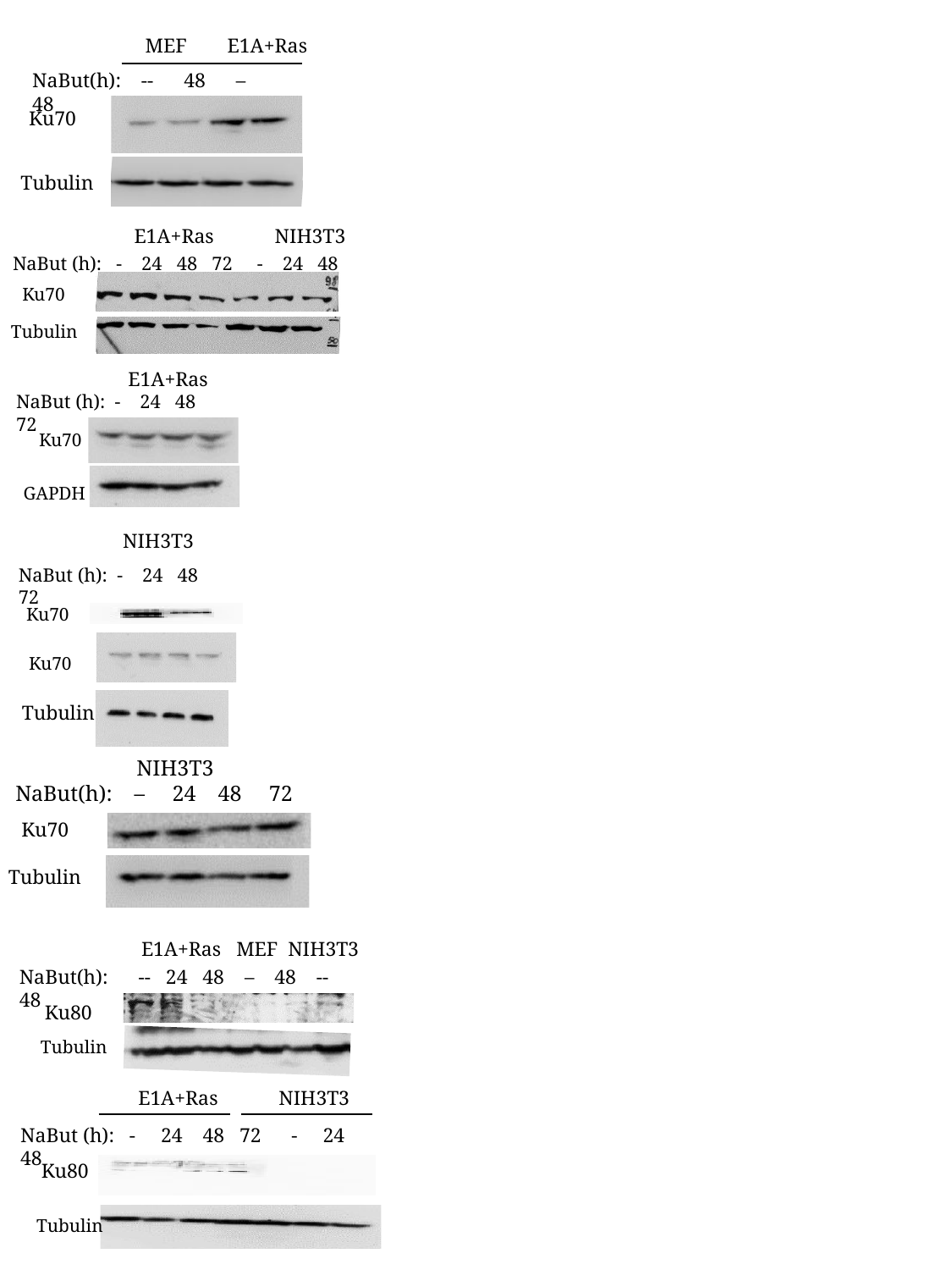

MEF E1A+Ras
NaBut(h): -- 48 – 48
Ku70
Tubulin
 E1A+Ras NIH3T3
NaBut (h): - 24 48 72 - 24 48
Ku70
Tubulin
 E1A+Ras
NaBut (h): - 24 48 72
Ku70
GAPDH
NIH3T3
NaBut (h): - 24 48 72
Ku70
Ku70
Tubulin
 NIH3T3
NaBut(h): – 24 48 72
Ku70
Tubulin
 E1A+Ras MEF NIH3T3
NaBut(h): -- 24 48 – 48 -- 48
Ku80
Tubulin
 E1A+Ras NIH3T3
NaBut (h): - 24 48 72 - 24 48
Ku80
Tubulin

## Slide 6
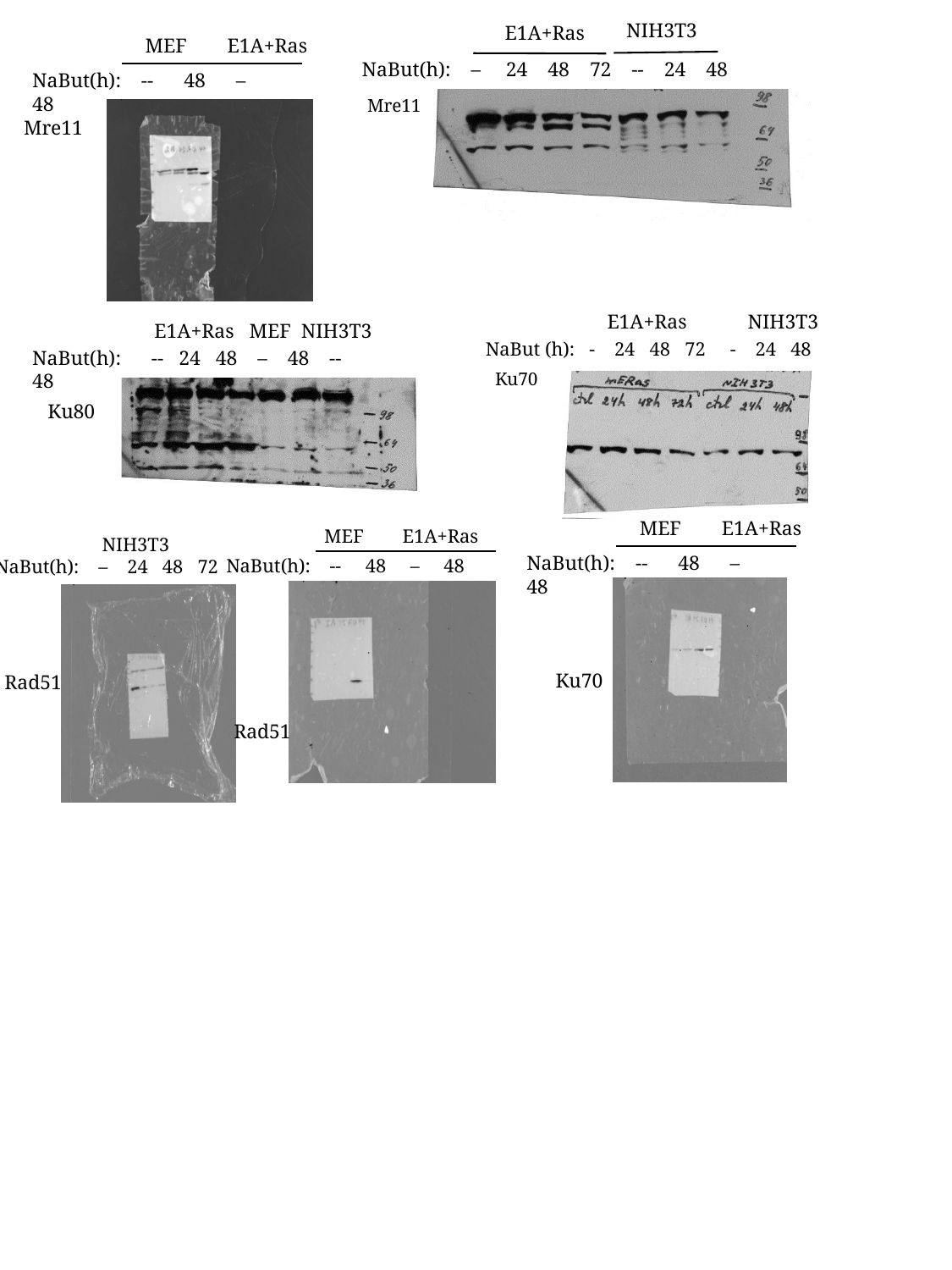

NIH3T3
E1A+Ras
 MEF E1A+Ras
NaBut(h): – 24 48 72 -- 24 48
NaBut(h): -- 48 – 48
Mre11
Mre11
 E1A+Ras NIH3T3
 E1A+Ras MEF NIH3T3
NaBut (h): - 24 48 72 - 24 48
NaBut(h): -- 24 48 – 48 -- 48
Ku70
Ku80
 MEF E1A+Ras
 MEF E1A+Ras
 NIH3T3
NaBut(h): – 24 48 72
NaBut(h): -- 48 – 48
NaBut(h): -- 48 – 48
Ku70
Rad51
Rad51
